# Altered Expression of Dual‐Specificity Phosphatases in Macrophages Exposed to Lipid Nanoparticles

**DOI:** 10.1096/fba.2026-00055

**Published:** 2026-05-21

**Authors:** David M. Cauvi, Dennis Hawisher, Ikenna Aniegbuna, Julia Derunes, Antonio De Maio

**Affiliations:** ^1^ Division of Trauma, Critical Care, Burns, and Acute Care Surgery, Department of Surgery, School of Medicine University of California San Diego La Jolla California USA; ^2^ Maximizing Access to Research Careers (MARC Program) University of California San Diego La Jolla California USA

**Keywords:** inflammation, lipid nanoparticles, phosphatases, phospholipids

## Abstract

Acute inflammation is rapidly elicited in response to pathogens, toxins, and other cellular components that interact with immune cells through surface receptors, activating signaling cascades that lead to the expression of molecules aimed at neutralizing the pathogen and stimulating other cells to begin the healing process. However, a robust inflammatory response can be detrimental if it is prolonged. Therefore, the inflammatory response must be tightly regulated to prevent secondary harmful effects. In this regard, cells contain molecular switches that turn the inflammatory process on and off. The rapid response to infection and injury is mediated by constitutive cellular proteins that are activated by posttranslational modifications, of which phosphorylation is the most common. Consequently, compensatory mechanisms often involve the removal of phosphate groups by phosphatases. A family of phosphatases known as dual‐specificity phosphatases (DUSPs), which dephosphorylate both tyrosine and serine/threonine residues, has emerged as a critical mechanism for controlling inflammation. To date, more than 40 DUSPs have been identified in the human genome. Despite their shared catalytic specificity, they appear to participate in diverse cellular processes and are expressed in response to various stimuli. In the present study, we detected a distinctive pattern of DUSP expression in response to exogenous lipid nanoparticles and toxins. These observations suggest that the transcriptional regulation of several DUSPs may be coordinated. They also raise the possibility that DUSPs may play redundant roles to ensure the proper resolution of the inflammatory response.

## Graphical abstract

Rapid responses to infection and tissue injury rely on constitutively expressed proteins that are activated by posttranslational modifications, primarily phosphorylation. Feedback regulation involves phosphatases that remove phosphate groups, attenuating the response and constituting a cellular molecular off switch. Dual‐specificity phosphatases (DUSPs), which dephosphorylate both tyrosine and serine/threonine residues, are key regulators of inflammatory signaling. In the present study, we show a distinct DUSP expression pattern after exposure to liposomes and toxins, suggesting coordinated transcriptional regulation and potential functional redundancy in resolving the insult.

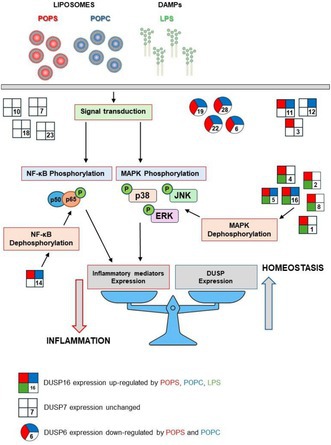

**Abbreviations**
DAMP: damage‐associated molecular patterns
DUSP: dual‐specificity phosphatases
ECV: extracellular vesicles
LNP: lipid nanoparticles
MKPs: mitogen‐activated protein kinase phosphatases
Mϕ: macrophage
PC: phosphatidylcholine
POPC: 1‐palmitoyl‐2‐oleoyl‐*sn*‐glycero‐3‐phosphocholine
POPS: 1‐palmitoyl‐2‐oleoyl‐*sn*‐glycero‐3‐phospho‐l‐serine

## 1 Introduction

Inflammation is a critical component of the response to infection and injury, which is triggered rapidly to control the harmful conditions. This process is initiated by the interaction of pathogens, toxins, and other cellular components, commonly referred to as damage‐associated molecular patterns (DAMPs), with surface receptors on immune cells, particularly macrophages and monocytes. These receptors, called pattern recognition receptors, activate a signaling cascade that ultimately leads to the activation of transcription factors responsible for the expression of inflammatory mediators such as cytokines [1]. However, inflammation must be tightly regulated because an excessive or prolonged activation is detrimental. Indeed, chronic inflammation is an underlying factor in many diseases [2]. The cellular signaling pathways activated during inflammation consist of numerous proteins regulated by reversible posttranslational modifications, of which phosphorylation is the most common [3]. Phosphorylation acts as a molecular switch that regulates gene expression at different levels [4]. Conversely, a compensatory mechanism that turns gene expression off is mediated by phosphatases [5, 6, 7]. These two processes, activation and deactivation, are temporally separated to ensure an appropriate response. Activation is mediated by kinases already constitutively present in cells, resulting in a rapid response. In contrast, attenuation of the response is slower and requires the transcription and translation of phosphatases. Thus, kinases and phosphatases act as “molecular switches” that regulate inflammation and other cellular processes.

The majority of signaling molecules involved in the inflammatory signaling are phosphorylated at tyrosine or serine/threonine residues. A family of phosphatases known as dual‐specificity phosphatases (DUSPs) can dephosphorylate both tyrosine and serine/threonine substrates [8]. Approximately 44 DUSPs have been identified in humans and 30 in mice [9, 10]. They are classified based on their target specificity and subcellular localization. Some DUSPs act on mitogen‐activated protein kinases (MAPKs), including DUSP1, DUSP2, DUSP4, DUSP5, DUSP6, DUSP7, DUSP8, DUSP9, DUSP10, and DUSP16, and are referred to as mitogen‐activated protein kinase phosphatases (MKPs), because they interact with kinases via a MAPK‐binding (KIM) domain at the N‐terminus [11, 12]. A second group, called atypical DUSPs, lack a MAPK‐binding domain, including DUSP3, DUSP11, DUSP12, DUSP13, DUSP14, DUSP15, DUSP18, DUSP19, DUSP21, DUSP22, DUSP23, DUSP26, DUSP27, and DUSP28. Several DUSPs are localized to the nucleus (DUSP1/MKP‐1, DUSP2, DUSP4/MKP‐2, DUSP5), others to the cytosol (DUSP6/MKP‐3, DUSP7/MKP‐X, DUSP9/MKP‐4), and some to both the nucleus and cytosol (DUSP8, DUSP10/MKP‐5, DUSP16/MKP‐7) [9, 12]. In general, DUSPs display short half‐lives, and their stability is regulated by posttranslational modifications such as ubiquitination and phosphorylation [13].

We have previously shown that peritoneal exposure of naïve mice to liposomes composed of palmitoyl‐oleoyl phosphatidylcholine (POPC) or palmitoyl‐oleoyl phosphatidylserine (POPS) induces the expression of several cytokines and chemokines [14, 15]. This strong inflammatory response associated with exposure to POPS liposomes was found to mitigate a septic insult [14]. Phospholipid liposomes are the basic components of lipid nanoparticles (LNPs), which have been used to deliver nucleic acid drugs [16, 17], and mRNA vaccines [18, 19]. A major advantage of using LNPs to deliver nucleic acids and other molecules is that the encapsulated material is protected from circulating degradative enzymes and other harmful agents. In addition, current LNPs can encapsulate material at high concentrations due to the presence of zwitterionic phospholipids. The primary phospholipid used in LNPs is phosphatidylcholine (PC), the most abundant cellular phospholipid. Cholesterol and polyethylene glycol are also added as stabilizing agents [20]. Phospholipids within LNPs have been considered inert components [21]. However, it could be argued that these natural molecules may play an independent biological role in modulating cellular responses [14, 15, 22]. A phospholipid membrane is also the structural basis of extracellular vesicles (ECVs) or exosomes, which play a role in intercellular communication [23] as well as in the disposal of damaged cellular components [24]. In the present study, we show that, in parallel with the induction of cytokines and chemokines by both POPS and POPC liposomes, there is also regulation of several DUSP genes, which are likely involved in controlling the inflammatory process.

## 2 Materials and Methods

### 2.1 Animals

CD‐1 [Crl:CD1 (ICR)] mice were obtained from Charles River Laboratories (Wilmington, MA, USA) and housed under specific pathogen‐free conditions at the University of California San Diego (UCSD) Animal Facility (La Jolla, CA, USA). Experiments were conducted using 9‐week‐old male. All procedures were approved by the UCSD Institutional Animal Care and Use Committee.

### 2.2 Cell Culture

RAW264.7 cell lines were obtained from ATCC (TIB‐67 and TIB‐71, respectively; ATCC, Manassas, VA, USA) and maintained in RPMI1640 with L‐glutamine and penicillin/streptomycin and supplemented with 10% FBS at 37°C.

### 2.3 Liposome Preparation

1‐Palmitoyl‐2‐oleoyl‐*sn*‐glycero‐3‐phospho‐l‐serine (POPS), 1‐Palmitoyl‐2‐oleoyl‐sn‐glycero‐3‐phosphocholine (POPC), and cholesterol in chloroform were all obtained from Avanti Polar Lipids (Alabaster, AL, USA). POPS and POPC in chloroform were dried under a nitrogen stream, and liposomes were prepared by rehydrating the dried lipid film (400 μg) in 120 μL of 50 mM endotoxin‐free Tris buffer (pH 7.4), with vortexing every 5 min for 30 min. The suspension was then extruded 15 times through a 100 nm membrane filter using a mini‐extruder apparatus (Avanti Polar Lipids). Subsequently, liposomes were centrifuged at 100,000 × *g* for 60 min at 4°C and resuspended in sterile PBS at a final concentration of 1 mg/mL. For cholesterol‐embedded POPC liposome experiments, 120 μg of cholesterol was combined with 280 μg of POPC in chloroform and processed following the same procedure as POPS and POPC liposomes. The size distribution and concentration of each liposome preparation were assessed using Nanoparticle Tracking Analysis (NTA, Nanosight NS300; Malvern Panalytical, UK). Endotoxin contamination was evaluated with the ToxinSensor Chromogenic LAL Endotoxin Assay Kit (GenScript, Piscataway, NJ, USA). The endotoxin concentration in POPC liposome preparations was typically below 0.01 EU/mL, corresponding to < 0.002 ng/mL of endotoxin.

### 2.4 Animal Liposome Treatment and Peritoneal Cell Isolation

CD‐1 mice were treated for 1 h with a 400 μg intraperitoneal (i.p.) injection of POPC or POPS liposomes. Control animals received an equal volume of PBS. Total peritoneal cells were then collected by peritoneal lavage. In brief, 5 mL serum‐free, phenol red–free RPMI 1640 was injected into the peritoneal cavity of CD‐1 mice, and, after gentle massage of the peritoneum to dislodge any loosely attached cells, the fluid was collected. Cell suspensions were immediately centrifuged for 10 min at 350 *g* and resuspended in PBS without Ca^2+^/Mg^2+^ supplemented with 0.5% bovine serum albumin for counting.

### 2.5 Cell Treatment

RAW264.7 Mϕs were incubated with equal amounts of POPC liposomes or POPC + 30% cholesterol (POPC + Chol) liposomes for 60 min. Control cells were treated under the same conditions in the absence of liposomes. In other experiments, RAW264.7 cells were treated with 100 ng/mL LPS for 60 min. Control cells received PBS vehicle only.

### 2.6 Global Gene Analysis by RNA Sequencing

Total peritoneal cells treated with POPC and POPS liposomes and RAW264.7 cells treated with POPC, POPC + Chol, or LPS were processed for global gene analysis by RNA sequencing. RNA was isolated with the RNeasy Mini Kit, according to the manufacturer's protocol (Qiagen, Germantown, MD, USA), and RNA integrity numbers were determined by the 4200 Tapestation (Agilent Technologies, Santa Clara, CA, USA). RNA sequencing libraries were then generated from 1 μg RNA with the TruSeq Stranded Total RNA Library Prep Gold Sample Prep Kit, according to the manufacturer's instructions (Illumina, San Diego, CA, USA), modifying the shear time to 5 min. RNA libraries were multiplexed and sequenced with 75‐bp single reads (SR75) to a depth of ∼40 million reads per sample on a HiSeq4000 (Illumina). Quality control of the raw fastq files was performed with the FastQC software tool. Sequencing reads were aligned to the mouse genome (mm10) with the Spliced Transcripts Alignment to a Reference (STAR), v.2.5.1a, sequence aligner (https://omictools.com/star‐3‐tool). Read quantification was performed with RNA‐Seq by Expectation Maximization (RSEM) v.1.3.0 (https://deweylab.github.io/RSEM) and GENCODE annotation (Mus_musculus.GRCm38.68.gtf) (https://www.gencodegenes.org/releases/current.html). The R BioConductor packages edgeR and limma were used to implement the limma‐voom method for differential expression analysis (https://www.bioconductor.org). The experimental design was modeled upon treatment (∼0 + treatment). Comparisons were made between the POPS‐PBS, POPC‐PBS, and POPS‐POPC groups for peritoneal cells and between POPC‐PBS, POPC + Chol‐PBS, and POPC/POPC + Chol for RAW264.7 cells. In addition, comparisons between LPS‐PBS were also made for RAW264.7 cells. In each bar graph, the expression of the indicated gene was shown as log‐transformed, normalized counts adjusted for the total read count per sample. Significance was defined by using an adjusted cutoff of *p* < 0.05 after multiple testing corrections with a moderated *t* statistic in limma. Functional enrichment of the differentially expressed genes was performed with the Bioconductor Gene Set Variation Analysis package for implementing Gene Set Enrichment Analysis (GSEA), Signaling Pathway Impact Analysis (SPIA) with the Bioconductor package SPIA. The data have been deposited in the National Center for Bioinformation Information (NCBI, Bethesda, MD, USA) Gene Expression Omnibus and are accessible through GEO series accession number GSE115489 (Total peritoneal cells treated with POPC and POPS liposomes); GSE319541 (Effect of free cholesterol on the inflammatory response induced by POPC liposomes).

### 2.7 Statistical Analysis

All data were analyzed using GraphPad Prism software (GraphPad Prism Software, San Diego, CA, USA). Significance was analyzed using one‐way ANOVA followed by Tukey's multiple comparison test or multiple unpaired Student's *t*‐test. A *p* < 0.05 value was considered statistically significant.

## 3 Results

### 3.1 POPS and POPC Liposomes Modulate the Expression of DUSPS in Peritoneal Macrophages

We previously reported that the delivery of liposomes composed of POPS or POPC into the peritoneal cavity of naïve mice were predominantly captured by resident macrophages, leading to changes in the expression of approximately 4104 and 1598 genes after exposure to POPS or POPC, respectively. This change in gene expression represented the modulation of about 40% of all genes analyzed [14, 15]. Pathway impact analysis revealed that a large proportion of these genes encoded inflammatory mediators, particularly cytokines and chemokines [9, 10]. We observed an overlap of genes whose expression was modulated by both phospholipids, as well as genes that were specifically affected by each lipid. These results were independently validated by measuring mRNA levels using quantitative RT‐PCR in isolated peritoneal macrophages. The increased expression of chemokines led to subsequent neutrophil infiltration into the peritoneal cavity, enabling the neutralization of bacterial infections [14, 15].

**FIGURE 1:**
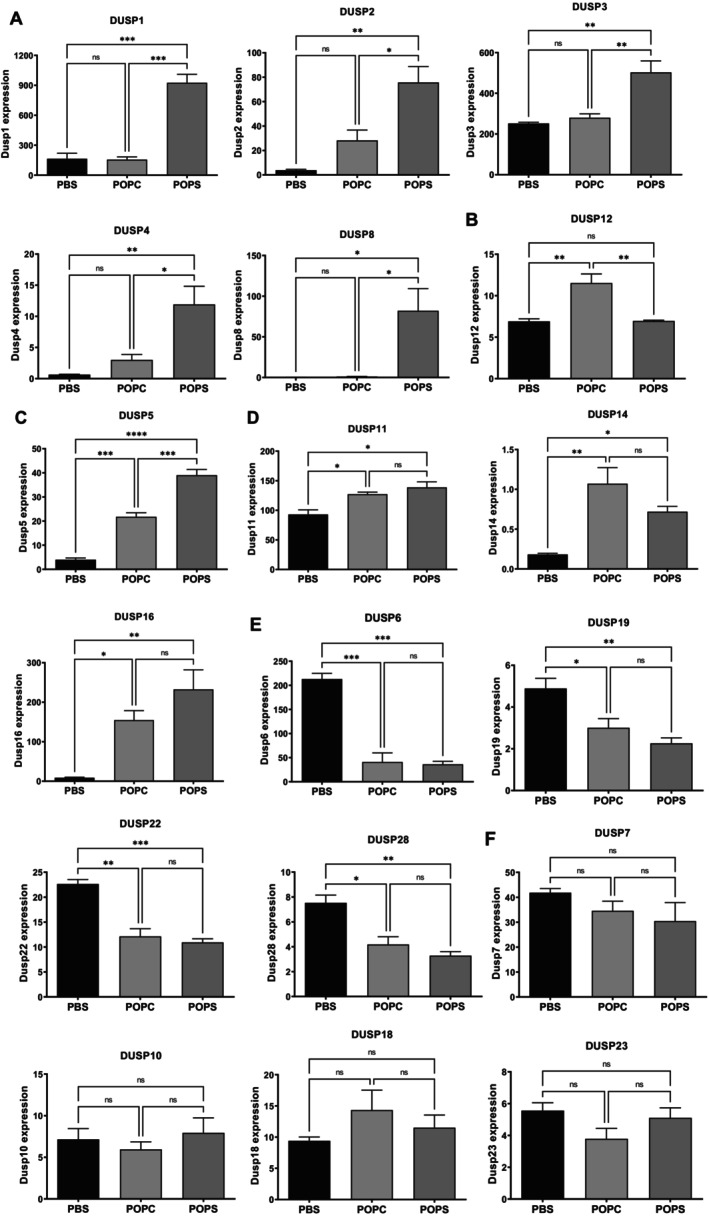
Expression of multiple DUSP family members in peritoneal macrophages is differentially modulated by POPC and POPS liposomes. CD‐1 mice (*n* = 3 per group) were injected intraperitoneally with POPC or POPS liposomes (400 μg) or an equivalent volume of PBS (vehicle control). Peritoneal cells were harvested 1 h postinjection and processed for RNA isolation followed by RNA‐seq analysis. DUSP gene expression was categorized as follows: (A) genes upregulated exclusively by POPS liposomes; (B) genes upregulated exclusively by POPC liposomes; (C) genes differentially upregulated by POPS and POPC liposomes; (D) genes similarly regulated by both POPS and POPC liposomes; (E) genes similarly downregulated by both POPS and POPC liposomes; (F) genes not affected by either POPS or POPC treatment. Results (*n* = 3) are expressed as means ± SEM, and statistical analysis was performed using one‐way ANOVA followed by Tukey's multiple comparison test with *, **, ***, and **** indicating *p* < 0.05, *p* < 0.01, *p* < 0.001, and *p* < 0.0001 respectively. The expression levels were presented as log‐transformed, normalized counts adjusted for the total read count per sample.

We found that the expression of several DUSPs was modulated after exposure to POPS and POPC liposomes. This observation called our attention because genes involved in inflammatory processes are activated rapidly mediated by posttranslational modifications, particularly phosphorylation, therefore, a counter‐regulatory response is expected to limit inflammation. In this context, the DUSP family of phosphatases has emerged as a key regulatory mediator that restrains the activation of signaling factors. We observed that DUSP1, DUSP2, DUSP3, DUSP4, and DUSP8 were upregulated exclusively by POPS liposomes (Figure 1A). In contrast, DUSP12 expression was elevated only after exposure to POPC liposomes (Figure 1B). DUSP5 levels were increased after incubation with either POPS or POPC compared with PBS controls but differed between the two phospholipids (Figure 1C). Both lipids increased expression of DUSP11, DUSP14, and DUSP16 (Figure 1D). Conversely, the basal levels of DUSP6, DUSP19, DUSP22, and DUSP28 were reduced after exposure to either POPS or POPC liposomes (Figure 1E). Finally, the expression of DUSP7, DUSP10, DUSP18, and DUSP23 was unaffected by either treatment (Figure 1F). These findings highlight the diversity and specificity of DUSP regulation following stimulation with pro‐inflammatory stimuli.

### 3.2 Incubation of Macrophages With LPS Also Increases DUSP Expression

**FIGURE 2:**
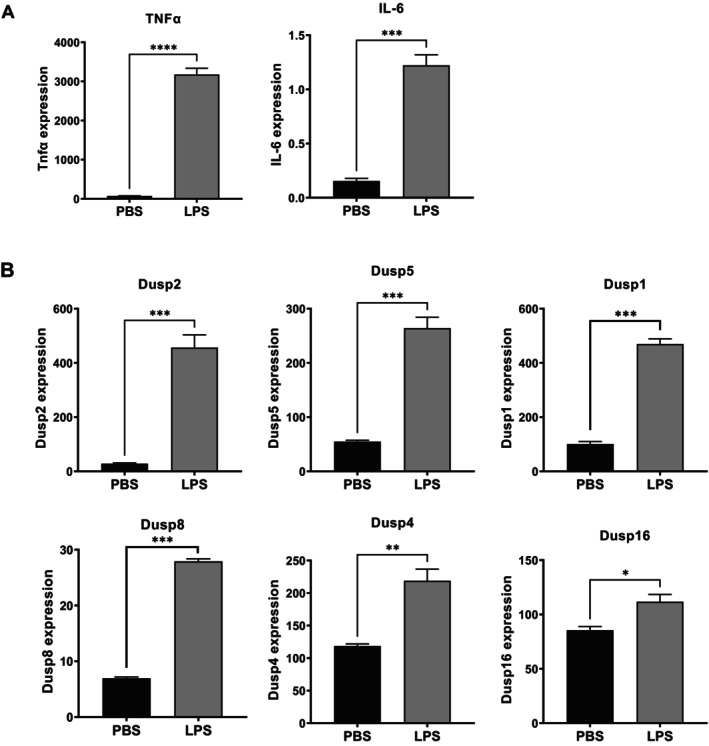
Expression of multiple DUSP family members is upregulated in macrophages by LPS treatment. Murine macrophages (RAW264.7 cells) were incubated with LPS for 1 h at 37°C and then lysed. Control cells were maintained under identical conditions without LPS. RNA was isolated and subjected to transcriptomic analysis. (A) LPS treatment upregulates TNF‐α and IL‐6 expression. (B) LPS treatment increases expression of multiple DUSP family members, including DUSP1, DUSP2, DUSP4, DUSP5, DUSP8, and DUSP16. Results (*n* = 3) are expressed as means ± SEM, and statistical analysis was performed using unpaired *t*‐test with *, **, ***, and **** indicating *p* < 0.05, *p* < 0.01, *p* < 0.001, and *p* < 0.0001 respectively. The expression levels were presented as log‐transformed, normalized counts adjusted for the total read count per sample.

To expand our prior observations, we analyzed DUSP expression after incubating murine cultured macrophages (RAW264 cells) with LPS, a well‐known inflammatory stimulus. RAW264 cells were incubated with LPS for 1 h at 37°C and lysed. Control cells were maintained under identical conditions without LPS exposure. Samples were subjected to transcriptomic analysis as described in Section 2. As expected, the expression of several cytokines, including TNF‐α and IL‐6, was elevated after LPS incubation compared with controls (Figure 2A), consistent with prior observations [25, 26]. Concurrently, the expression of several DUSPs (DUSP1, DUSP2, DUSP4, DUSP5, DUSP8, and DUSP16) was also increased (Figure 2B). Several other DUSPs were not affected by LPS treatment (Table 1). Notably, there was overlap between DUSPs induced by LPS and those induced by POPS liposomes (DUSP1, DUSP2, DUSP4, and DUSP8), as well as between LPS and both phospholipids (DUSP5 and DUSP16), suggesting that the modulation in the expression of these phosphatases is related to the inflammatory process rather than the specific stimuli.

**TABLE 1:** Change in Dusp genes' expression following LPS treatment.

Genes	logCPM	logFC	Fold change	Adj. *p*	Status
Dusp2	7.953	4.065	16.7	***	upReg
Dusp5	7.343	2.335	5.0	***	upReg
Dusp1	8.182	2.296	4.9	***	upReg
Dusp8	4.148	2.093	4.3	***	upReg
Dusp4	7.41	0.9551	1.9	**	upReg
Dusp16	6.632	0.4567	1.4	*	upReg
Dusp14	3.393	0.3869	1.3	NS	nonDE
Dusp12	2.919	−0.366	0.8	NS	nonDE
Dusp9	1.499	0.5242	1.4	NS	nonDE
Dusp18	4.373	−0.1914	0.9	NS	nonDE
Dusp11	7.39	−0.08711	0.9	NS	nonDE
Dusp3	7.97	−0.07077	1.0	NS	nonDE
Dusp22	4.477	0.08753	1.1	NS	nonDE
Dusp10	2.177	0.1399	1.1	NS	nonDE
Dusp28	1.911	−0.1372	0.9	NS	nonDE
Dusp6	4.554	0.06242	1.0	NS	nonDE
Dusp7	5.482	−0.02895	1.0	NS	nonDE
Dusp19	3.48	−0.03994	1.0	NS	nonDE

Abbreviations: nonDE, non‐differentially expressed; NS, nonsignificant; upReg, upregulated by LPS.

*p < 0.05; **p < 0.01; ***p < 0.001.

### 3.3 DUSP Expression Is Also Modulated by Incubation of RAW264 Cells With POPC Liposomes Containing or Lacking Free Cholesterol

**FIGURE 3:**
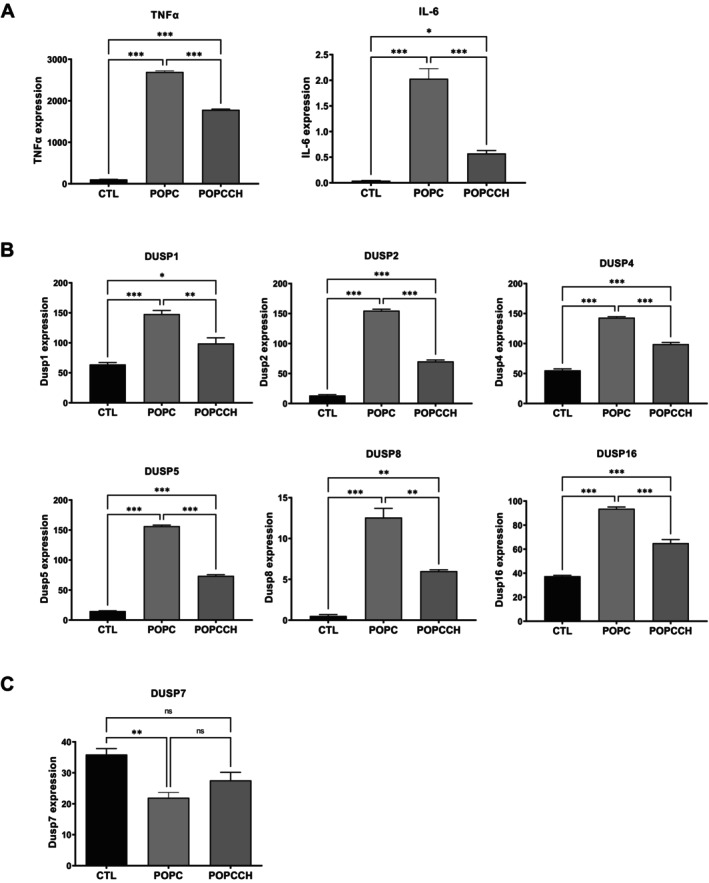
Cholesterol modulates POPC‐induced DUSP gene expression in macrophages. Murine macrophages (RAW264.7 cells) were left untreated (CTL) or treated with POPC liposomes (POPC) or POPC liposomes supplemented with cholesterol (POPCCH). Cells were harvested and processed for RNA isolation followed by transcriptomic analysis. (A) Incorporation of cholesterol into POPC liposomes reduces POPC‐induced TNF‐α and IL‐6 expression. (B) Cholesterol‐containing POPC liposomes attenuate POPC liposome‐induced expression of multiple DUSP family members. (C) Incorporation of cholesterol into POPC liposomes counteracts POPC‐induced downregulation of DUSP7 expression. Results (*n* = 3) are expressed as means ± SEM, and statistical analysis was performed using one‐way ANOVA followed by Tukey's multiple comparison test with *, **, and *** indicating *p* < 0.05, *p* < 0.01, and *p* < 0.001 respectively. The expression levels were presented as log‐transformed, normalized counts adjusted for the total read count per sample.

RAW264 cells were also incubated with POPC liposomes under culture conditions. Similar to our observations in vivo, cytokine expression increased after POPC liposome exposure compared with controls (Figure 3A). However, the addition of free cholesterol to POPC liposomes reduced cytokine expression (Figure 3A), consistent with our previous findings [26].

The levels of six DUSPs (DUSP1, DUSP2, DUSP4, DUSP5, DUSP8, and DUSP16) increased upon incubation with POPC liposomes (Figure 3B), whereas DUSP7 expression was reduced (Figure 3C). When free cholesterol was added to POPC liposomes, the same pattern of elevated DUSP expression relative to control was observed; however, these levels were reduced compared with POPC liposomes lacking cholesterol (Figure 3B). Thus, the effects of free cholesterol on DUSP expression mirrors the reduction on cytokine expression after exposure to POPC liposomes with the sterol (Figure 3A). The expression of other DUSPs was not affected by incubation with POPC liposomes (Table 2). Indeed, these observations suggest a link between cytokine and DUSP expression during inflammation.

**TABLE 2:** Change in Dusp genes' expression following POPC treatment.

Genes	logCPM	logFC	Fold change	Adj. *p*	Status
Dusp5	5.797	3.398	10.5	***	upReg
Dusp2	5.708	3.624	12.3	***	upReg
Dusp4	6.524	1.347	2.5	***	upReg
Dusp16	5.932	1.331	2.5	***	upReg
Dusp1	6.604	1.184	2.3	***	upReg
Dusp8	1.72	4.626	24.7	***	upReg
Dusp7	4.793	−0.7074	0.6	**	downReg
Dusp10	1.249	0.7132	1.6	NS	nonDE
Dusp28	2.016	−0.5488	0.7	NS	nonDE
Dusp6	4.449	−0.1719	0.9	NS	nonDE
Dusp12	3.701	−0.1532	0.9	NS	nonDE
Dusp22	4.543	−0.07786	0.9	NS	nonDE
Dusp18	3.893	−0.07357	1.0	NS	nonDE
Dusp14	3.072	−0.07133	1.0	NS	nonDE
Dusp19	3.486	−0.04851	1.0	NS	nonDE
Dusp3	8.03	−0.01141	1.0	NS	nonDE
Dusp9	1.775	0.01763	1.0	NS	nonDE
Dusp11	7.263	−0.0004513	1.0	NS	nonDE

Abbreviations: downReg, downregulated by POPC; nonDE, non‐differentially expressed; NS, nonsignificant; upReg, upregulated by POPC.

**p < 0.01; ***p < 0.001.

### 3.4 The Expression of Other Phosphatases Is Also Upregulated After Exposure to POPC and POPS Liposomes

**FIGURE 4:**
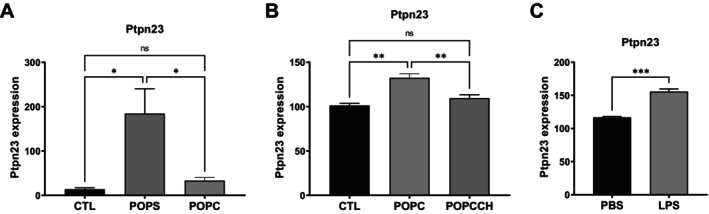
Expression of the non‐receptor tyrosine phosphatase Ptpn23 is modulated by POPS and POPC liposomes, with or without cholesterol, as well as by LPS treatment. (A) CD‐1 mice (*n* = 3 per group) were injected intraperitoneally with POPC or POPS liposomes (400 μg) or an equivalent volume of PBS (vehicle control). Peritoneal cells were harvested 1 h postinjection and processed for RNA isolation followed by RNA‐seq analysis. Ptpn23 expression is selectively induced by POPS, but not by POPC, liposomes. (B) Murine macrophages (RAW264.7 cells) were left untreated (CTL) or treated with POPC liposomes (POPC) or POPC liposomes supplemented with cholesterol (POPCCH). Cells were harvested and processed for RNA isolation followed by transcriptomic analysis. Cholesterol‐containing POPC liposomes inhibit POPC liposome‐induced expression of Ptpn23. (C) Murine macrophages (RAW264.7 cells) were incubated with LPS for 1 h at 37°C and then lysed. Control cells were maintained under identical conditions without LPS. RNA was isolated and subjected to transcriptomic analysis. LPS treatment upregulates Ptpn23 expression. Results (*n* = 3) are expressed as means ± SEM, and statistical analysis was performed either using one‐way ANOVA followed by Tukey's multiple comparison test with * and ** indicating *p* < 0.05 and *p* < 0.01 respectively (A, B) or unpaired *t*‐test with *** indicating *p* < 0.001 (C). The expression levels were presented as log‐transformed, normalized counts adjusted for the total read count per sample.

**FIGURE 5:**
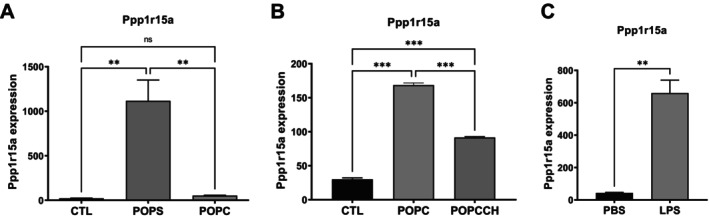
Expression of the protein phosphatase 1 regulatory subunit Ppp1r15a is strongly induced in vivo by POPS liposomes, and in vitro by POPC liposomes with or without cholesterol, as well as by LPS treatment. (A) CD‐1 mice (*n* = 3 per group) were injected intraperitoneally with POPC or POPS liposomes (400 μg) or an equivalent volume of PBS (vehicle control). Peritoneal cells were harvested 1 h postinjection and processed for RNA isolation followed by RNA‐seq analysis. Ppp1r15a expression is selectively induced by POPS, but not by POPC, liposomes. (B) Murine macrophages (RAW264.7 cells) were left untreated (CTL) or treated with POPC liposomes (POPC) or POPC liposomes supplemented with cholesterol (POPCCH). Cells were harvested and processed for RNA isolation followed by transcriptomic analysis. Incorporation of cholesterol into POPC liposomes significantly attenuated POPC liposome‐induced expression of Ppp1r15a. (C) Murine macrophages (RAW264.7 cells) were incubated with LPS for 1 h at 37°C and then lysed. Control cells were maintained under identical conditions without LPS. RNA was isolated and subjected to transcriptomic analysis. LPS treatment strongly upregulates Ppp1r15a expression. Results (*n* = 3) are expressed as means ± SEM, and statistical analysis was performed either using one‐way ANOVA followed by Tukey's multiple comparison test with ** and *** indicating *p* < 0.01 and *p* < 0.001 respectively (A, B) or unpaired *t*‐test with ** indicating *p* < 0.01 (C). The expression levels were presented as log‐transformed, normalized counts adjusted for the total read count per sample.

As part of our transcriptome analysis, we investigated whether the expression of other phosphatases was modulated upon incubation with POPC or POPS liposomes. Phosphatases fall into two main families that remove phosphate from either tyrosine or serine/threonine residues. We examined these groups and found that expression of the non‐receptor tyrosine phosphatase *Ptpn23* increased 13.1‐fold after POPS exposure in peritoneal macrophages in vivo, but not after incubation with POPC liposomes in vitro (Figure 4A). In contrast, a modest increase in *Ptpn23* expression was observed in RAW264 cells after POPC treatment, which was reduced when free cholesterol was included in the liposomes (Figure 4B). *Ptpn23* expression also increased after LPS stimulation of RAW264 cells (Figure 4C). The expression of another tyrosine phosphatase non‐receptor, *Ptpn1*, was increased 4.9‐fold after incubation with POPC liposomes and 2.3‐fold after exposure to POPS liposomes on peritoneal macrophages. The expression of *Ptpn2* and *Ptpn12* were also increased after incubation with POPS liposome in 2‐ and 2.7‐fold, respectively. Exposure to RAW264 cells to LPS increased the expression of *Ptpn14* in 2.1‐fold (Table 3). PPP1R15A, a regulatory subunit of protein phosphatase 1 (PP1) and a key component of the ER stress response, also known as growth arrest and DNA damage‐inducible protein 34 (GADD34), was strongly induced. *Ppp1r15a* expression increased 2.4‐ and 43.1‐fold after incubation of peritoneal macrophages with POPC or POPS liposomes, respectively (Figure 5A). Its expression also increased 5.6‐fold in RAW264 cells exposed to POPC but was downregulated when free cholesterol was included in the POPC liposomes (Figure 5B). LPS treatment significantly induced *Ppp1r15a* expression (Figure 5C). The expression of *Ptpre* and *Ptprj* increased respectively 2.1‐ and 2‐fold after POPS stimulation while *Ptprj* increased 1.9‐fold after POPC exposure in peritoneal macrophages (Tables 4 and 5).

**TABLE 3:** Change in Ptpn genes' expression following LPS treatment.

Genes	logCPM	logFC	Fold change	Adj. *p*	Status
Ptpn23	7.096	0.4838	1.4	***	upReg
Ptpn14	1.794	1.082	2.1	***	upReg
Ptpn18	5.145	−0.3042	0.8	NS	nonDE
Ptpn4	6.925	0.1351	1.1	NS	nonDE
Ptpn7	7.328	0.1174	1.1	NS	nonDE
Ptpn1	6.114	0.1244	1.1	NS	nonDE
Ptpn11	7.345	0.07515	1.1	NS	nonDE
Ptpn6	7.912	−0.0979	0.9	NS	nonDE
Ptpn9	6.201	0.09085	1.1	NS	nonDE
Ptpn22	7.455	0.03609	1.0	NS	nonDE
Ptpn2	5.716	0.06086	1.0	NS	nonDE
Ptpn21	5.451	−0.03296	1.0	NS	nonDE
Ptpn12	6.59	−0.01559	1.0	NS	nonDE
Ptpn5	1.878	−0.02291	1.0	NS	nonDE

Abbreviations: nonDE, non‐differentially expressed; NS, nonsignificant; upReg, upregulated by LPS.

***p < 0.001.

**TABLE 4:** Change in Ptpr genes' expression following POPS treatment in vivo.

Genes	logCPM	logFC	Fold change	Adj. *p*	Status
Ptpre	7.193	1.082	2.1	**	upReg
Ptprj	9.606	0.9949	2.0	**	upReg
Ptpro	6.522	0.5807	1.5	*	upReg
Ptprm	4.712	0.6931	1.6	*	upReg
Ptprc	9.638	0.6439	1.6	NS	nonDE
Ptprcap	4.733	−0.5433	0.7	NS	nonDE
Ptprs	5.448	0.563	1.5	NS	nonDE
Ptprg	1.149	1.135	2.2	NS	nonDE
Ptprf	0.664	0.4529	1.4	NS	nonDE
Ptpru	−0.3244	0.5963	1.5	NS	nonDE
Ptpra	6.596	−0.06207	1.0	NS	nonDE

Abbreviations: nonDE, non‐differentially expressed; NS, nonsignificant; upReg, upregulated by POPS.

*p < 0.05 ; **p < 0.01.

**TABLE 5:** Change in Ptpr genes' expression following POPC treatment in vivo.

Genes	logCPM	logFC	Fold change	Adj. *p*	Status
Ptprj	9.606	0.9317	1.9	*	upReg
Ptprc	9.638	0.7011	1.6	NS	nonDE
Ptpra	6.596	−0.4463	0.7	NS	nonDE
Ptpru	−0.3244	1.221	2.3	NS	nonDE
Ptpre	7.193	0.2731	1.2	NS	nonDE
Ptpro	6.522	−0.1563	0.9	NS	nonDE
Ptprm	4.712	−0.09946	0.9	NS	nonDE
Ptprs	5.448	−0.1508	0.9	NS	nonDE
Ptprcap	4.733	0.08126	1.1	NS	nonDE
Ptprg	1.149	−0.2479	0.8	NS	nonDE
Ptprf	0.664	−0.02878	1.0	NS	nonDE

Abbreviations: nonDE, non‐differentially expressed; NS, nonsignificant; upReg, upregulated by POPC.

*p < 0.05.

## 4 Discussion

Like Newton's third law, “every action has an equal and opposite reaction,” a similar principle can be applied to biology: a response to stress is accompanied by a compensatory reaction. This principle is particularly true for the inflammatory response, which is followed by an anti‐inflammatory counterpart. If inflammation is not controlled, it can become harmful to the organism. Indeed, chronic inflammation, an underlying condition in many diseases [2]. Inflammation is triggered by exogenous cellular stimuli such as infectious agents, tissue damage, and other stressors, currently referred to as DAMPs. These molecules interact with surface receptors and activate signaling cascades that lead to the expression of inflammatory mediators such as cytokines [1]. This signaling process is rapidly activated through posttranslational modifications of target molecules, mainly by kinase activities [3]. Since these substrates remain active as long as they are phosphorylated, they must be dephosphorylated to attenuate the process. Based on these assumptions, it is expected that activation of inflammation is followed by the expression of specific phosphatases that mitigate the response.

Indeed, a family of enzymes known as dual‐specificity phosphatases (DUSPs) has been identified as part of the anti‐inflammatory process [9, 10, 11, 12]. Their name derives from their ability to remove phosphate groups from tyrosine, serine, and threonine residues on target molecules. To date, approximately 30–40 DUSPs have been identified in humans and rodents [9, 11, 12]. They target cellular kinases such as members of the mitogen‐activated protein kinase (MAPK) family, which play crucial roles in inflammation, cell proliferation, survival, and death. DUSPs were previously referred to as MAPK phosphatases (MKPs). They remove phosphate groups from threonine and/or tyrosine residues from the T‐X‐Y motif within the kinase active site [8]. Some DUSPs bind directly to MAPKs via a KIM domain at the N‐terminal region of the phosphatase and are termed *typical* DUSPs. Others lack this domain and are classified as *atypical* phosphatases [11, 12]. DUSPs have been associated with a variety of disease conditions, including infection [11, 27], cancer [10, 28], asthma and chronic obstructive pulmonary disease [29, 30], obesity [31], and autoimmunity [32]. Moreover, DUSPs may be involved in tumor development and progression, with increased expression reported in several human tumors, positioning them as potential cancer therapeutic targets [28].

We have previously found that the phospholipids POPC and POPS, when formulated into liposomes, activate an inflammatory response. Phospholipid liposomes are the basic components of lipid nanoparticles (LNPs), which have gained increasing attention as carriers for mRNA vaccines and nucleic acid therapeutics [16, 17, 18, 19]. Although LNP contains several other components including ionizable phospholipids and cholesterol, phospholipids like PC are necessary for the formation of the particle [16, 33]. Indeed, phospholipids spontaneously assemble into vesicles through entropic processes [34], protecting their luminal cargo from degradation by external enzymes. Although phospholipids within LNPs were once considered inert components, accumulating evidence indicates that they play active biological roles [14, 15, 22]. For example, phospholipids within LNP in vaccine may act as adjuvants [35, 36]. Phospholipid membranes also represent an essential component of extracellular vesicles (ECVs) or exosomes, which are small vesicles encapsulated by a lipid bilayer. ECVs play key roles in intercellular communication by carrying cargo that maintains homeostasis or activates responses to stress or danger [23]. Our prior studies have shown that POPC and POPS liposomes activate cytokine and chemokine expression both in vivo (peritoneal cells) and in macrophages cultured conditions [14, 15, 25]. Although POPC and POPS liposomes share overlapping activities, they trigger responses through different mechanisms. POPS liposomes bind to the cell‐surface scavenger receptor CD36, which recognizes the negative charge of the phospholipid, and initiates a signaling cascade that activates NF‐κB and promotes TNFα transcription. In contrast, POPC liposomes are internalized by pinocytosis rather than receptor binding, activating the inflammatory response from within endosomes [25].

We found that several DUSPs were stimulated by POPS (DUSP1, 2, 3, 4, and 8), by POPC (DUSP12) liposomes, or by both (DUSP5, 11, 14, and 16). In contrast, expression of DUSP6, 19, 22, and 28 was reduced by POPC or POPS liposomes, and other DUSPs (7, 10, 18, and 23) were unaffected. Interestingly, the expression of DUSP1, 2, 4, 5, 8, and 16 also increased when RAW264.7 cells were incubated with POPC liposomes in culture conditions, and the same DUSPs were upregulated after LPS exposure to RAW264.7 cells. These findings suggest that a specific subset of DUSPs is activated during the inflammatory response regardless of the initiating stimulus. Thus, the transcriptional activity of these DUSPs may be triggered by similar factors activating the expression of cytokines via NF‐κB. We also speculate that these DUSPs may provide synergistic functions or that their expression is redundant to ensure timely control of inflammation. Furthermore, their expression may be coordinated to compensate for the reduced activity of any single member. Indeed, reduced DUSP2 expression in knockout mice resulted in increased DUSP1 levels, suggesting crosstalk between DUSPs [27].

We have previously shown that adding free cholesterol to POPC liposomes attenuates the inflammatory response, at least at the level of TNFα production, an effect not observed with esterified cholesterol [26]. Therefore, we investigated whether complementing POPC liposomes with free cholesterol affects DUSP expression. The same phosphatases stimulated by POPC liposomes alone were reduced by the addition of free cholesterol, although their expression was not completely abolished. Notably, the downregulation pattern produced by free cholesterol was similar between cytokines and DUSPs, suggesting their expression may be aligned. This raises the possibility that a common signaling molecule responsible for cytokine induction may also coordinate DUSP expression. The effect of free cholesterol on inflammation is complex and may involve changes in membrane fluidity. Moreover, cholesterol could be extracted from liposome‐loaded endosomes by the NPC1/2 system and transported to other cellular membranes [37]. If delivered to the ER, cholesterol may be oxidized to oxysterols that activate peroxisome proliferator–activated receptors (PPARs) and liver X receptors (LXRα/LXRβ), both known regulators of inflammation [38, 39]. Indeed, LXRs have also been shown to negatively regulate NF‐κB signaling [38, 40, 41]. Thus, the reduction in DUSP expression by cholesterol may compromise regulation of the inflammatory response and could contribute to atherosclerosis.

Previous studies have shown that several DUSPs were upregulated following LPS stimulation, including DUSP1 [42, 43, 44, 45], DUSP4 [27], DUSP5 [46], and DUSP10 [47]. In contrast, other DUSPs, including DUSP3, DUSP6, DUSP7, DUSP11, DUSP12, DUSP14, DUSP18, DUSP22, and DUSP26 were unaffected by LPS stimulation [9, 45]. Moreover, increased DUSP1 expression correlated with reduced cytokine production and improved resistance to LPS challenge in mice [42, 48]. Overexpression of DUSP5 also reduced cytokine levels after LPS stimulation, whereas deletion of DUSP5 has the opposite effect [46]. Similarly, DUSP1 deletion enhanced cytokine production and susceptibility to endotoxic shock [42, 48]. Deletion of DUSP11 resulted in overproduction of LPS‐induced cytokines and increased susceptibility to endotoxic shock [49]. In contrast, DUSP2‐null mice show reduced cytokine expression after LPS exposure and improved survival following septic challenge [27, 44]. This improved survival correlated with reduced cytokine expression, consistent with the role of cytokine storms in poor sepsis outcomes [50, 51]. Although these observations appear to contradict the concept that DUSPs universally act as negative regulators of inflammation, they can be reconciled by a compensatory upregulation of DUSP1 in DUSP2‐null mice [27]. DUSP1 expression is also increased by extracellular HSP70, which reduced inflammation in airway epithelial cells [30]. Notably, extracellular HSP70 itself can activate inflammatory responses [52, 53].

We also found that several tyrosine‐specific phosphatases are upregulated following POPC liposome stimulation (*Ptpn4*, 12, and 23), whereas *Ptpn6* levels are downregulated. *Ptpn14* and *Ptpn23* are also increased in RAW264.7 cells after LPS incubation. Although information on the effects of LPS on *Ptpn* expression or function is limited, several *Ptpns* have been associated with inflammatory diseases [54, 55]. Their expression is also increasingly linked to cancer incidence [56, 57, 58].

In summary, the balance between rapid activation of the inflammatory response and the timely ability to halt the process is critical for maintaining health and restoring cellular homeostasis. DUSPs have emerged as important molecular switches that mediate a “switch‐off” mechanism of inflammation that may be critical in avoiding prolonged inflammation, which has detrimental consequences for human health, as well as chronic inflammation that underlies many diseases. Our observations may add a new dimension to the biology of DUSPs. The finding that a specific set of DUSPs is expressed in tandem suggests that their transcriptional regulation may be coordinated or that they perform redundant functions. It is also possible that individual DUSPs within this set target distinct components of the signaling pathway to ensure proper resolution of the inflammatory response. These ideas may open new avenues for investigating the functional roles of these phosphatases.

## Author Contributions

David M. Cauvi and Antonio De Maio conceived and designed the research; David M. Cauvi, Dennis Hawisher, Julia Derunes, and Ikenna Aniegbuna performed the research and acquired the data; David M. Cauvi and Antonio De Maio analyzed and interpreted the data; David M. Cauvi and Antonio De Maio wrote the manuscript and all authors were involved in drafting and revising the manuscript.

## Funding

This study was supported by University of California, San Diego Academic Senate Grant #RG114348, 2024.

## Conflicts of Interest

The authors declare no conflicts of interest.

## Data Availability

All the data that support the findings of this study are available in the manuscript, including methods.
